# Disability Status and Induction Into US Medical Honor Societies

**DOI:** 10.1001/jamanetworkopen.2024.54180

**Published:** 2025-01-09

**Authors:** Mytien Nguyen, Samantha L. Schroth, Karina Pereira-Lima, Christopher J. Moreland, Sarwat I. Chaudhry, Amy N. Addams, Lisa M. Meeks, Dowin B. Boatright

**Affiliations:** 1 Department of Immunobiology, Yale School of Medicine, New Haven, Connecticut; 2 Department of Pathology, Northwestern Feinberg Medical School, Chicago, Illinois; 3 Department of Neurology, University of Michigan Medical School, Ann Arbor; 4 Department of Internal Medicine, Dell Medical School at the University of Texas at Austin; 5 Section of General Internal Medicine, Department of Medicine, Yale School of Medicine, New Haven, Connecticut; 6 Association of American Medical Colleges, Washington, DC; 7 Department of Family Medicine, University of Michigan Medical School, Ann Arbor; 8 Department of Emergency Medicine, New York University Grossman School of Medicine, New York

## Abstract

This cohort study examines disability status and membership in medical honor societies among medical students in the US.

## Introduction

Membership in prestigious medical honor societies, such as Alpha Omega Alpha (AΩA) and the Gold Humanism (GHHS), is associated with success in medicine.^1^ Studies have demonstrated inequities in honor society membership by students’ race, ethnicity, socioeconomic status, and sexual orientation.^2,3^ However, little is known about the association of disability status with AΩA and GHHS membership.

## Methods

This cohort study followed the Strengthening the Reporting of Observational Studies in Epidemiology (STROBE) reporting guideline. It was deemed exempt by the institutional review board of the University of Michigan. Informed consent was not required because data were deidentified. We analyzed deidentified data in May 2024 from the Association of American Medical Colleges Graduation Questionnaire (GQ) for MD graduates in academic years 2020 to 2022. Race and ethnicity were self-reported and recategorized into 8 groups: American Indian, Alaska Native, Native Hawaiian, or Pacific Islander; Asian; Black; Hispanic; White; other; and multiracial. Self-reported disability status were categorized into nondisabled, cognitive, motor or sensory, and chronic illness categories. Students reporting more than 1 disability type were categorized as more than 1 disability. Our study cohort included graduates who took both the 1991 and 2015 Medical College Admission Test (MCAT). Since the 1991 and 2015 MCAT examinations are distinct and to minimize bias, quartiles were computed for each MCAT examination, and a final MCAT quartile variable was created by combining the quartiles of the 1991 and 2015 MCAT.

Multivariate logistic regression was used to estimate the adjusted odds of induction into honor society by disability status, adjusting for MCAT score quartiles, sex, race, and ethnicity due to known association with honor society induction.^2,3^ Analyses were performed using Stata 18.0 (StataCorp). Data were analyzed from May to August 2024.

## Results

Among 50 139 students who completed the GQ, 40 600 students (90.0%) attended schools with both AΩA and GHHS (students at schools with AΩA election in the senior year were excluded), 3849 students (7.7%) had missing demographic and MCAT data and were excluded. Among 36 751 students in the study cohort, 19 352 students were female (52.7%), 7653 students were Asian (20.8%), 2122 students were Black (5.8%), 3622 students were Hispanic (9.9%), 20 825 students were White (56.7%), and 3132 were students with disabilities (8.5%) (Table 1).

**Table 1.** Association of Disability Status With Induction Into Alpha Omega Alpha and Gold Humanism Honor Society

Characteristic	Total (N = 36 751)	Alpha Omega Alpha		Gold Humanism	
		No. (%)	aOR (95% CI)	No. (%)	aOR (95% CI)
Disability status					
None	33 619	7233 (21.5)	1 [Reference]	5175 (15.3)	1 [Reference]
Yes	3132	354 (11.3)	0.47 (0.41-0.52)	425 (13.5)	0.83 (0.75-0.93)
Sex					
Male	17 399	3486 (20)	1 [Reference]	2073 (11.9)	1 [Reference]
Female	19 352	4101 (21.1)	1.22 (1.15-1.28)	3527 (18.2)	1.62 (1.53-1.72)
Race or ethnicity					
American Indian, Alaska Native, Native Hawaiian, Pacific Islander	65	7 (10.7)	0.44 (0.20-0.98)	12 (18.4)	1.24 (0.66-2.34)
Asian	7653	1248 (16.3)	0.53 (0.50-0.57)	1046 (13.6)	0.87 (0.81-0.94)
Black or African American	2122	228 (10.7)	0.50 (0.43-0.58)	468 (22.0)	1.48 (1.32-1.66)
Hispanic	3622	484 (13.3)	0.58 (0.53-0.65)	552 (15.2)	1.00 (0.90-1.11)
White	20 825	5097 (24.4)	1 [Reference]	3113 (14.9)	1 [Reference]
Multiracial	1381	301 (21.7)	0.85 (0.74-0.97)	214 (15.4)	1.01 (0.87-1.18)
Other	1083	222 (20.4)	0.83 (0.71-0.97)	195 (18.0)	1.26 (1.07-1.48)
MCAT, quartile					
First (lowest)	10 906	1469 (13.4)	1 [Reference]	1792 (16.4)	1 [Reference]
Second	10 148	1989 (19.5)	1.45 (1.34-1.56)	1599 (15.7)	1.02 (0.95-1.10)
Third	8661	2132 (24.6)	1.98 (1.84-2.14)	1242 (14.3)	0.96 (0.88-1.04)
Fourth (highest)	7036	1997 (28.3)	2.44 (2.25-2.64)	967 (13.7)	0.95 (0.87-1.04)

Abbreviations: aOR, adjusted odds ratio; MCAT, Medical College Admission Test.

Compared with nondisabled peers, a lower proportion of students with disabilities were inducted into AΩA (11.3% vs 21.5%; aOR, 0.47; 95% CI, 0.41-0.52). Students with disabilities were less likely to be inducted into GHHS (13.5% vs 15.4%; aOR, 0.83; 95% CI, 0.75-0.93). The gap in induction into AΩA for students with disabilities was larger than that for GHHS. Students with cognitive disability (11.0%; aOR, 0.46; 95% CI, 0.40-0.53), motor or sensory disability (15.1%; aOR, 0.64; 95% CI, 0.42-0.96), chronic illness (11.3%; aOR, 0.43; 95% CI, 0.30-0.60), and those with more than 1 disability (8.6%; aOR, 0.31; 95% CI, 0.18-0.54) had lower rates of AΩA induction compared with nondisabled students (21.5%). Compared with students who were not disabled, students with cognitive disability were significantly less likely to be inducted into GHHS (aOR, 0.77 [95% CI, 0.68-0.87]) (Table 2).

**Table 2.** Association of Disability Type With Induction Into Alpha Omega Alpha and Gold Humanism Honor Society

Characteristic	Total (N = 36 751)	Alpha Omega Alpha		Gold Humanism	
		No. (%)	aOR (95% CI)^1^	No. (%)	aOR (95% CI)^1^
Disability type					
No	33 604	7236 (21.5)	1 [Reference]	5183 (15.4)	1 [Reference]
Cognitive disability	2453	270 (11.0)	0.46 (0.40-0.53)	310 (12.6)	0.77 (0.68-0.87)
Motor or sensory	185	28 (15.1)	0.64 (0.42-0.96)	36 (19.4)	1.35 (0.93-1.95)
Chronic illness	335	38 (11.3)	0.43 (0.30-0.60)	48 (14.3)	0.83 (0.61-1.13)
More than 1 type	174	15 (8.6)	0.31 (0.18-0.54)	23 (13.2)	0.75 (0.48-1.17)
Sex					
Male	17 399	3486 (20.0)	1 [Reference]	2073 (11.9)	1 [Reference]
Female	19 352	4101 (21.1)	1.22 (1.16-1.28)	3527 (18.2)	1.62 (1.53-1.72)
Race or ethnicity					
American Indian, Alaska Native, Native Hawaiian, Pacific Islander	65	7 (10.7)	0.44 (0.20-0.98)	12 (18.4)	1.25 (0.66-2.35)
Asian	7653	1248 (16.3)	0.53 (0.50-0.57)	1046 (13.6)	0.87 (0.81-0.94)
Black or African American	2122	228 (10.7)	0.50 (0.43-0.58)	468 (22.0)	1.48 (1.32-1.66)
White	20 825	5097 (24.4)	1 [Reference]	3113 (14.9)	1 [Reference]
Hispanic	3622	484 (13.3)	0.58 (0.53-0.65)	552 (15.2)	1.00 (0.91-1.11)
Multiracial	1381	301 (21.7)	0.85 (0.74-0.97)	214 (15.4)	1.01 (0.87-1.18)
Other	1083	222 (20.4)	0.83 (0.71-0.96)	195 (18.0)	1.26 (1.07-1.48)
MCAT, quartile					
First (lowest)	10 906	1469 (13.4)	1 [Reference]	1792 (16.4)	1 [Reference]
Second	10 148	1989 (19.5)	1.45 (1.34-1.56)	1599 (15.7)	1.02 (0.94-1.10)
Third	8661	2132 (24.6)	1.99 (1.84-2.15)	1242 (14.3)	0.96 (0.88-1.04)
Fourth (highest)	7036	1997 (28.3)	2.44 (2.25-2.64)	967 (13.7)	0.95 (0.87-1.04)

Abbreviations: aOR, adjusted odds ratio; MCAT, Medical College Admission Test.

## Discussion

Compared with students who were not disabled, students with disabilities were half as likely to be inducted into AΩA, and 17% less likely to be inducted into GHHS. Students reporting cognitive disability, chronic illnesses, and multiple disabilities experienced the lowest honor society induction rates. These findings build upon prior literature which demonstrated higher burnout risk and lower placement into graduate medical education among students with disabilities compared with their peers.^4^ Inequity in honor society selection for students with disabilities may stem from structural and interpersonal bias in medical education, resulting in suboptimal learning environment for students with disabilities.^5^ Stigma remains a significant concern for students with disabilities, presenting significant barriers to disclosure and accommodations.^6^ These disparities could have significant implications for the diversity of the physician workforce and warrant additional investigation.

Limitations include lack of other characteristics and academic achievements not accounted for in the study that may contribute to student’s induction into AΩA and GHHS. Study results highlight the persisting necessity to address inequities in honor society membership and the need to build equity in medical education using an antiableist and antioppressive lens.

## Notes

Supplement.
Data Sharing Statement
